# An integrated autophagy-related gene signature predicts prognosis in human endometrial Cancer

**DOI:** 10.1186/s12885-020-07535-4

**Published:** 2020-10-27

**Authors:** Jun Zhang, Ziwei Wang, Rong Zhao, Lanfen An, Xing Zhou, Yingchao Zhao, Hongbo Wang

**Affiliations:** 1 Department of Obstetrics and Gynecology, Union Hospital, Tongji Medical College, Huazhong University of Science and Technology, Wuhan, Hubei China; 2 Department of Cancer Center, Union Hospital, Tongji Medical College, Huazhong University of Science and Technology, Wuhan, Hubei China

**Keywords:** Prognosis, Autophagy, Endometrial cancer, Molecular biomarkers, The Cancer genome atlas

## Abstract

**Background:**

Globally, endometrial cancer is the fourth most common malignant tumor in women and the number of women being diagnosed is increasing. Tumor progression is strongly related to the cell survival-promoting functions of autophagy. We explored the relationship between endometrial cancer prognoses and the expression of autophagy genes using human autophagy databases.

**Methods:**

The Cancer Genome Atlas was used to identify autophagy related genes (ARGs) that were differentially expressed in endometrial cancer tissue compared to healthy endometrial tissue. Gene Ontology and Kyoto Encyclopedia of Genes and Genomes were referenced to identify important biological functions and signaling pathways related to these differentially expressed ARGs. A prognostic model for endometrial cancer was constructed using univariate and multivariate Cox, and Least Absolute Shrinkage and Selection Operator regression analysis. Endometrial cancer patients were divided into high- and low-risk groups according to risk scores. Survival and receiver operating characteristic (ROC) curves were plotted for these patients to assess the accuracy of the prognostic model. Using immunohistochemistry the protein levels of the genes associated with risk were assessed.

**Results:**

We determined 37 ARGs were differentially expressed between endometrial cancer and healthy tissues. These genes were enriched in the biological processes and signaling pathways related to autophagy. Four ARGs (CDKN2A, PTK6, ERBB2 and BIRC5) were selected to establish a prognostic model of endometrial cancer. Kaplan–Meier survival analysis suggested that high-risk groups have significantly shorter survival times than low-risk groups. The area under the ROC curve indicated that the prognostic model for survival prediction was relatively accurate. Immunohistochemistry suggested that among the four ARGs the protein levels of CDKN2A, PTK6, ERBB2, and BIRC5 were higher in endometrial cancer than healthy endometrial tissue.

**Conclusions:**

Our prognostic model assessing four ARGs (CDKN2A, PTK6, ERBB2, and BIRC5) suggested their potential as independent predictive biomarkers and therapeutic targets for endometrial cancer.

**Supplementary information:**

**Supplementary information** accompanies this paper at 10.1186/s12885-020-07535-4.

## Background

Autophagy is the process by which cells engulf their cytoplasmic proteins and organelles, envelop them into vesicles, and combine with lysosomes to form autolysosomes in order to renew intracellular components [1–3]. Autophagy is closely associated with the occurrence and development of various diseases, such as malignant tumors, infectious diseases, and autoimmune diseases [4–8]. However, the role of autophagy in tumors has not been fully elucidated. It is generally believed that in the initial stage of tumor formation, autophagy inhibits the growth of malignant tumors; once malignant tumors are formed, autophagy promotes the development of cancer [9, 10]. Many studies have attempted to clarify the role and complex functionalities of autophagy in malignant tumors with the intent of suppressing the development of malignancies by upregulating or inhibiting autophagy [11].

Endometrial cancer is one of the most common malignant tumors in women. It ranks fourth among the European and North American women, accounting for approximately 6% of new cancer cases and 3% of cancer deaths every year; most of the incidences occur during the perimenopausal and menopausal periods [12]. The treatment of early endometrial cancer is mainly surgical and has good prognoses. Unfortunately, recurrence and metastasis occur in some patients, for whom radiotherapy, chemotherapy, or a combination both is required; however, outcomes are poor [13–16].

The relationship between autophagy and the occurrence and development of endometrial cancer has been reported in the early studies, for example, autophagy-mediated regulation of the adaptive response to targeted therapy [17]. Previous studies have mostly focused on the role of autophagy in the development and treatment of endometrial cancer [18–20]. However, there are only a few large-scale data mining studies that have analyzed the effects of autophagy genes on the progression and prognosis of endometrial cancer. In this study, we analyzed the transcriptomic data of endometrial cancer samples downloaded from The Cancer Genome Atlas (TCGA) public database and constructed a prognostic model for endometrial cancer with four autophagy related genes (ARGs), which could accurately assess the prognostic risk of patients with endometrial cancer.

## Methods

### Patient information and databases

Transcript information of endometrial cancer samples was downloaded from TCGA database (https://portal.gdc.cancer.gov/), and the clinical information on these patients was downloaded from the UCSC Xena database (https://xenabrowser.net/heatp/). A total of 222 autophagy genes were obtained from the human autophagy database (http://www.autophagy.lu/).

### Screening and enrichment analysis of differentially expressed genes

The Limma package in R statistical software was used to filtrate differentially expressed genes associated with autophagy (FDR Filter = 0.05,logFC filter = 1). The ClusterProfiler, org. Hs.eg.db, and ggplot2 packages in R were applied to perform enrichment analysis of all the differentially expressed ARGs using the Gene Ontology (GO) and Kyoto Encyclopedia of Genes and Genomes (KEGG) databases in order to discover the main biological characteristics of these genes [21].

### Construction of prognostic models

We performed a univariate Cox regression analysis of ARGs, which were differentially expressed in endometrial cancer. Univariate Cox regression analyses were plotted using the “survival” package; a *P* value less than 0.05 was considered a significant difference threshold. In order to simplify the parameters of the model and avoid overfitting of the model, we carried out the Least Absolute Shrinkage and Selection Operator (LASSO) regression analysis using the “glmnet” R package [22]. Multivariate Cox regression analysis was carried out for the genes obtained by LASSO regression, and those with a 95% confidence interval for a hazard ratio (HR) > 1 or < 1 were selected as parameters for the final prognostic model. The expression values of all prognostic genes were multiplied by their regression coefficients, which eventually added up to a risk score for each sample. Survival curves were plotted with the “survminer” R package, and a receiver operating characteristic (ROC) curve evaluating the accuracy of the prognostic model was constructed using the “timeROC” R package [23].

### Training and validation of the prognostic model

The TCGA-Uterine Corpus Endometrial Carcinoma (UCEC) cohort was randomly split into a training set and a verification set using the “caret” R package, and the ratio was set to 1:1. We obtained a training set with 270 samples and verification set with 270 samples. The training set was used to perform multivariate Cox regression to obtain the parameters of the prognostic model, and the verification set was used to confirm the prediction accuracy of this prognostic model. The risk score of each sample in both sets was calculated and the samples were divided into a high-risk group and a low-risk group, using the median risk score as the division between high- and low-risk groups.

### Immunohistochemical data of four risk ARGs

The Human Protein Atlas (HPA, https://www.proteinatlas.org/) is a database that contains a large amount of human protein data, which can help us understand the protein expression levels of specific genes in different tissues [24]. We downloaded immunohistochemical pictures of four risk ARGs in normal endometrium and endometrial cancer from this website and we calculated the ISH score of each sample (ISH score = staining intensity * staining area score). There are four levels of staining intensity: negative, 0; weak, 1; moderate, 2; and strong, 3. These correspond to five levels of staining area score: < 5%, 0; 5 to 25%, 1; 5 to 50%, 2; 50 to 75%, 3; and > 75%, 4.

### Statistical analysis

All statistical analyses were performed using the R software (v.3.6.1. https://www.r-project.org/) and GraphPad Prism software (v.8.01; GraphPad Software, San Diego, CA, USA). Univariate Cox regression, multivariate Cox regression, and LASSO regression were used to establish the prognostic model. Kaplan-Meier plotter and ROC curve were used to evaluate the accuracy of the prognostic model. An unpaired t-test was used to compare the ISH score between the normal endometrial tissue and UCEC tissue. *P* < 0.05 was considered statistically significant.

## Results

### Differentially expressed ARGs

**Fig. 1:**
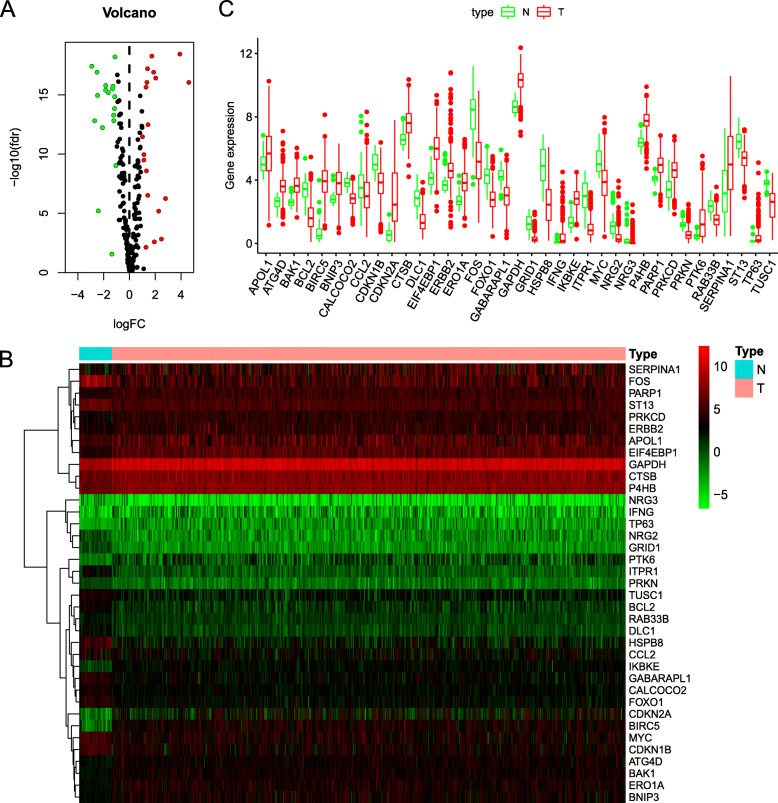
Differentially expressed ARGs. **a** The volcano plot for the 222 autophagy-related genes from the TCGA data portal. Red represents high expression, and green represents low expression. **b** Hierarchical clustering of differentially expressed ARGs expression levels. **c** The expression patterns of 37 autophagy-related genes in endometrial cancer and normal endometrial tissues. Red columns represent tumor tissue, and green columns represent normal tissue. The height of the column represents its expression in the corresponding sample

We analyzed the expression of 222 ARGs in 552 endometrial cancer tissues and 35 non-tumor tissues using the Wilcoxon signed-rank test in R. We obtained 37 differentially expressed ARGs, according to the criteria of |log2FC| > 1 and FDR < 0.05. These ARGs include 19 up-regulated genes (PARP1, PRKCD, CTSB, APOL1, ATG4D, BNIP3, BAK1, P4HB, ERBB2, ERO1A, GAPDH, IKBKE, TP63, EIF4EBP1, SERPINA1, IFNG, PTK6, BIRC5, and CDKN2A) and 18 downregulated genes (ITPR1, FOS, GRID1, HSPB8, NRG3, NRG2, DLC1, BCL2, FOXO1, CCL2, PRKN, TUSC1, CDKN1B, GABARAPL1,ST13, RAB33B, CALCOCO2, and MYC). The volcano map (Fig. 1a), heatmap (Fig. 1b), and boxplot (Fig. 1c) were visualized for these ARGs (Supplementary Table S1).

### GO enrichment analysis of differentially expressed ARGs

**Fig. 2:**
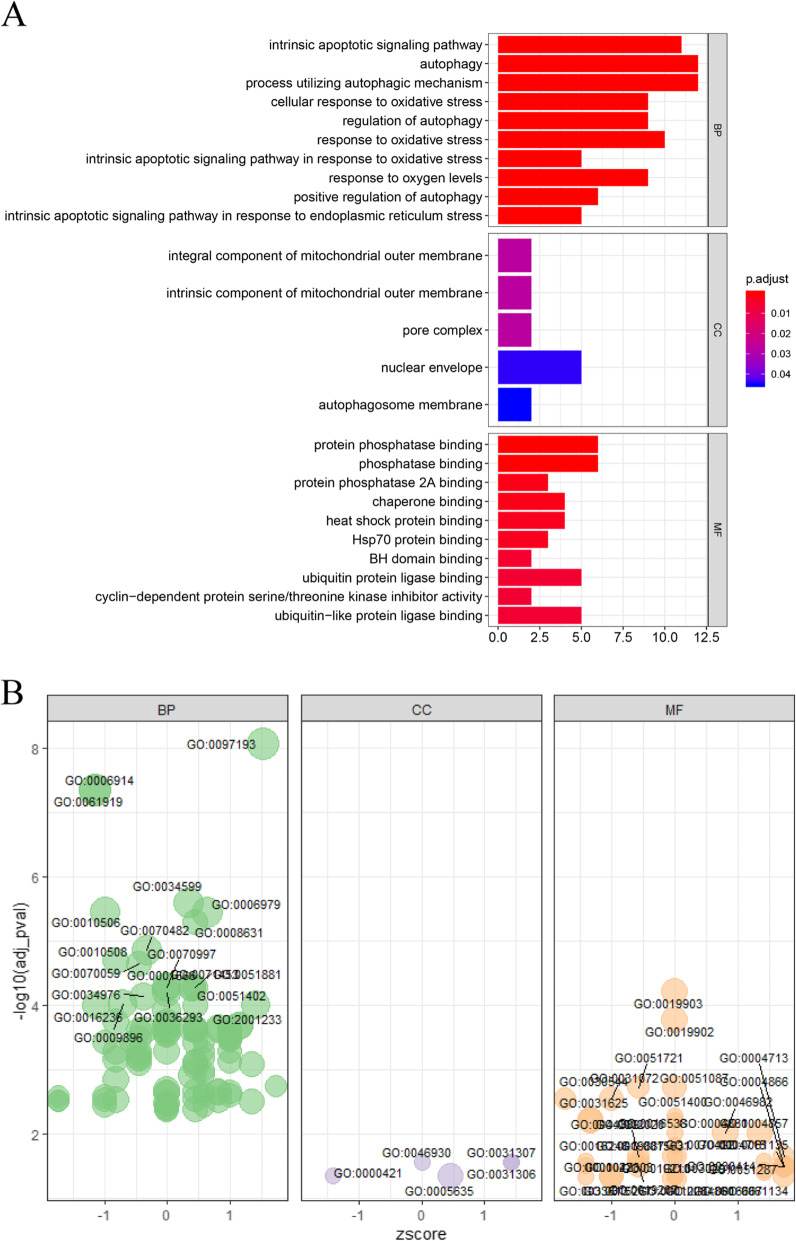
GO enrichment analysis of differentially expressed ARGs. **a** Bar plot of significant GO terms, on the left is the name of the GO term, the length of the histogram on the right indicates the number of genes contained, and the color indicates the adjusted *P*-value. **b** Bubble plot of enriched GO terms. The Z-score is plotted on the x-axis, and the -log (adjusted p-value) is plotted on the y-axis. The size of the bubble reflects the number of genes enriched in the term. BP means “Biological process”; MF means “Molecular function”; CC means “Cellular component”

GO functional enrichment analysis of these 37 differentially expressed ARGs was performed (Supplementary Table S2), and the enrichment results were visualized to understand the biological functions of these genes. The results showed these ARGs were primarily involved with the intrinsic apoptotic signaling pathway, processes utilizing the autophagic mechanism, the cellular response to oxidative stress, integral components of the mitochondrial outer membrane, autophagosome membrane, protease binding, and heat shock protein binding (Fig. 2a, b).

### KEGG enrichment analysis of differentially expressed ARGs

**Fig. 3:**
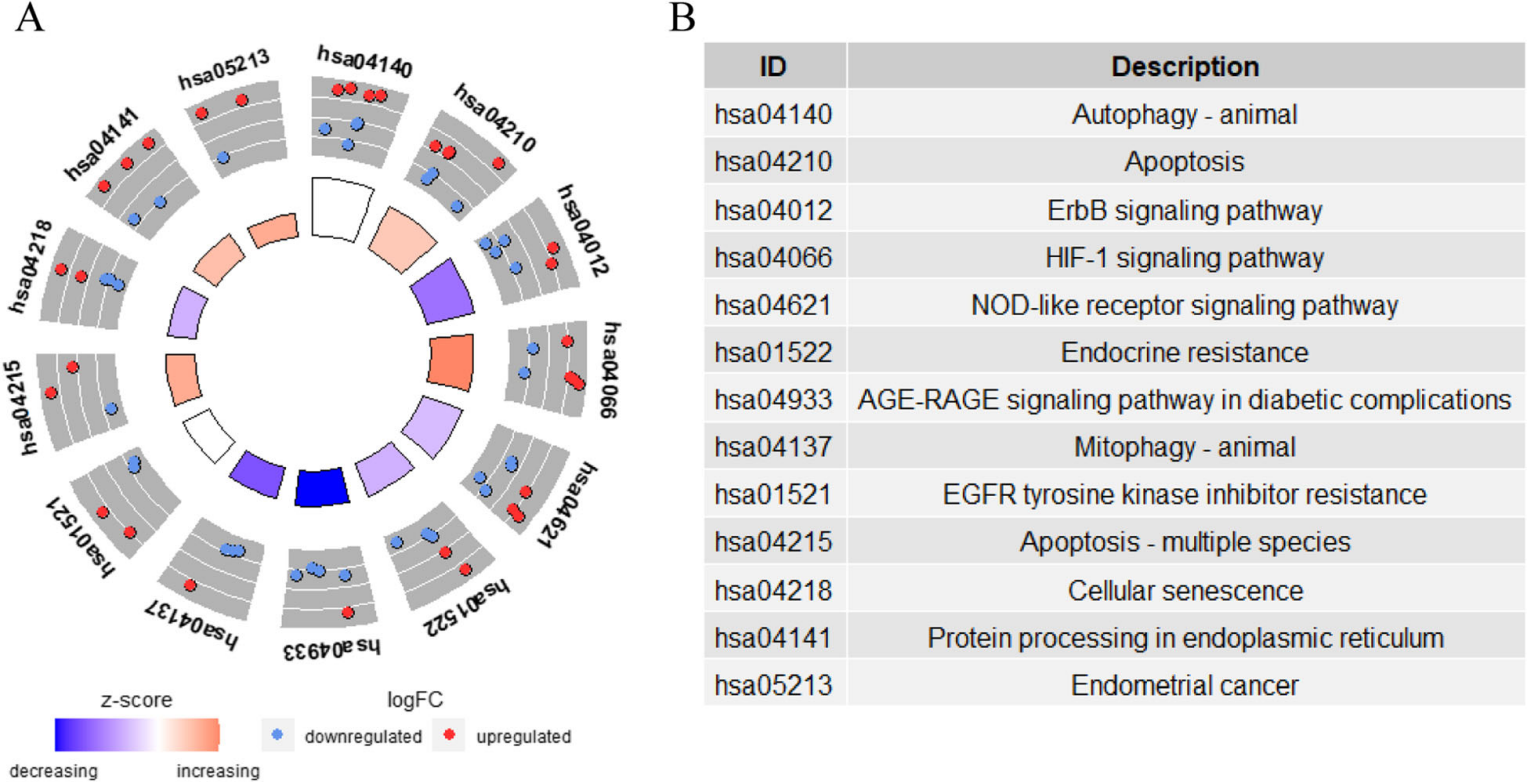
KEGG enrichment analysis of differentially expressed ARGs. **a** Circle plot of KEGG enrichment analysis, each independent trapezoidal area represents a KEGG pathway, where red dots represent genes that are up-regulated, and blue represent downregulated genes. **b** The table lists the name of each KEGG term

The results of the KEGG pathway enrichment analysis indicated that the differentially expressed ARGs were related to autophagy, apoptosis, the ErbB signaling pathway, the HIF-1 signaling pathway, cellular senescence, the AGE-RAGE signaling pathway in diabetic complications, protein processing in the endoplasmic reticulum, endometrial cancer, the FoxO signaling pathway, and the estrogen signaling pathway (Fig. 3, Table 1). In endometrial cancer, three differentially expressed ARGs (ERBB2, BAK1 and MYC), which are closely related to the occurrence of endometrial cancer, were increased. The estrogen signaling pathway, which is highly associated with the development of endometrial cancer, showed enrichment of four differentially expressed ARGs (ITPR1, FOS, PRKCD, and BCL2).

**Table 1:** KEGG enrichment results for differentially expressed ARGs

ID	Description	P	geneID
hsa04140	Autophagy - animal	7.80E−06	ITPR1/CTSB/PRKCD/ ATG4D/GABARAPL1/ BNIP3/RAB33B/ BCL2
hsa04210	Apoptosis	4.99E−05	PARP1/ITPR1/FOS/CTSB/BAK1 /BCL2/BIRC5
hsa04012	ErbB signaling pathway	4.99E−05	CDKN1B/NRG2/ERBB2/MYC/EIF4EBP1/NRG3
hsa04066	HIF-1 signaling pathway	1.62E−04	CDKN1B/ERBB2/IFNG/BCL2/GAPDH/EIF4EBP1
hsa04621	NOD-like receptor signaling pathway	1.94E−04	ITPR1/CTSB/PRKCD/GABARAPL1/CCL2/BCL2/IKBKE
hsa01522	Endocrine resistance	8.02E−04	CDKN1B/FOS/ERBB2/CDKN2A/BCL2
hsa04933	AGE-RAGE signaling pathway in diabetic complications	8.02E−04	CDKN1B/PRKCD/FOXO1/CCL2/BCL2
hsa04137	Mitophagy - animal	1.84E−03	CALCOCO2/PRKN/GABARAPL1/BNIP3
hsa01521	EGFR tyrosine kinase inhibitor resistance	2.62E−03	NRG2/ERBB2/BCL2/EIF4EBP1
hsa04215	Apoptosis - multiple species	2.62E−03	BAK1/BCL2/BIRC5
hsa04218	Cellular senescence	3.49E−03	ITPR1/FOXO1/CDKN2A/MYC/EIF4EBP1
hsa04141	Protein processing in endoplasmic reticulum	3.57E−03	ERO1A/PRKN/BAK1/BCL2/P4HB
hsa05213	Endometrial cancer	9.22E−03	ERBB2/BAK1/MYC
hsa04068	FoxO signaling pathway	1.00E−02	CDKN1B/FOXO1/GABARAPL1/BNIP3
hsa04915	Estrogen signaling pathway	1.17E−02	ITPR1/FOS/PRKCD/BCL2

### Survival-related ARGs and the prognostic model

**Fig. 4:**
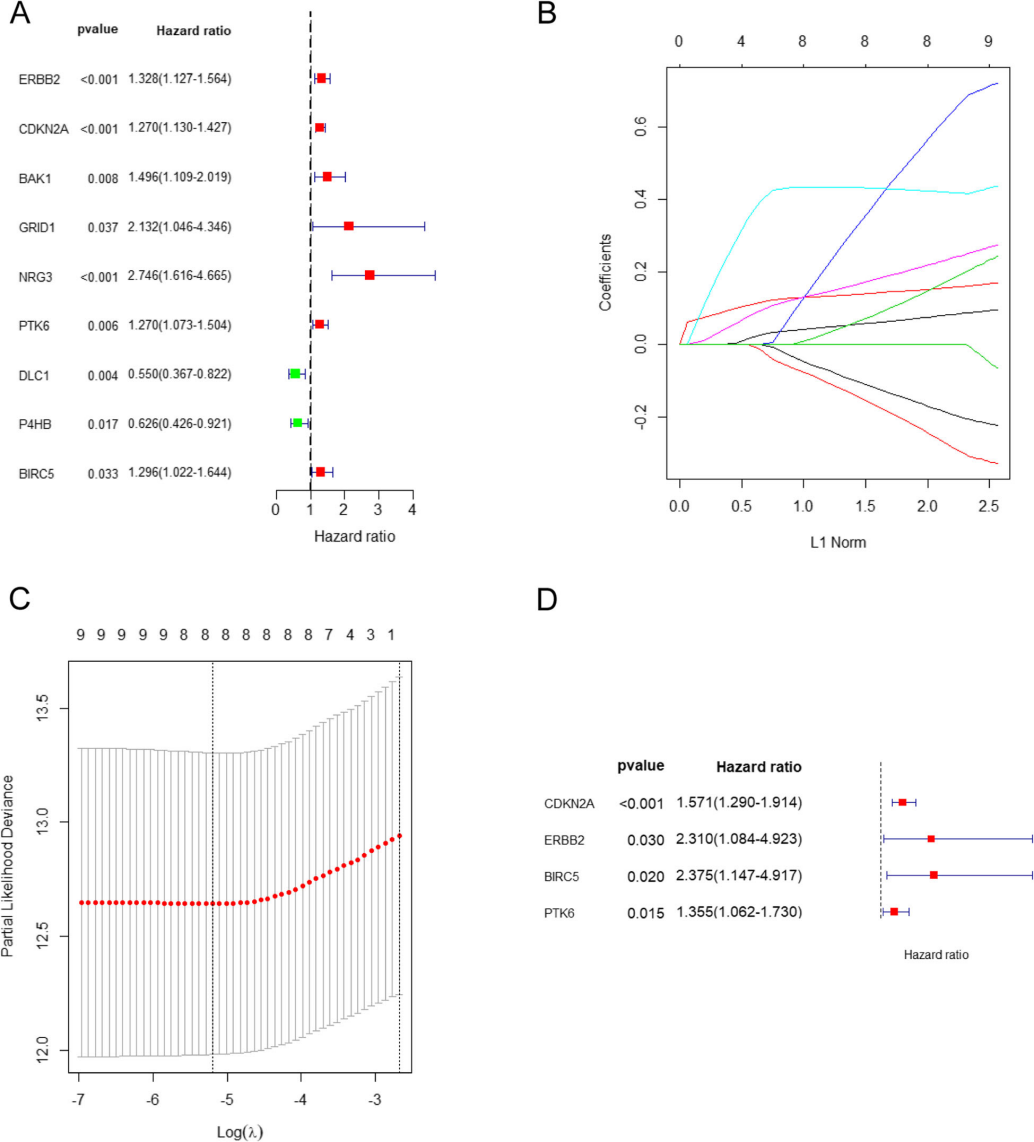
Survival-related ARGs and the prognostic model. **a** Forest plots visualizing the HRs of 9 prognostic ARGs identified by univariate Cox analysis of TCGA UCEC, protective associations are shown in green, and risk factors are shown in red; **b** LASSO coefficient profiles of the 9 prognostic ARGs, each curve represents a coefficient; as λ changes, the coefficient enters the prognostic model with a non-zero value. **c** Cross-validation to select the optimal tuning parameter (λ), the first black dotted line indicates the optimal number of parameters of the multivariate risk prognosis model. **d** Forest plots visualizing the HRs of 4 prognostic ARGs identified by multivariate Cox analysis of training cohort

We performed univariate Cox regression of 37 differentially expressed ARGs, and 9 ARGs associated with endometrial cancer prognosis were obtained, including ERBB2, CDKN2A, BAK1, GRID1, NRG3, PTK6, DLC1, P4HB, and BIRC5 (*P* < 0.05). Seven prognosis-related ARGs (ERBB2, CDKN2A, BAK1, GRID1, NRG3, PTK6, and BIRC5) were considered risk factors (the minimum value of the 95% CI was greater than 1), and their high expression indicates a poor prognosis. Conversely, the high expression of the remaining two genes (DLC1 and P4HB) indicates better survival. Results were visualized using a forest plot (Fig. 4a).

LASSO regression analysis was then conducted to exclude genes that may be highly correlated with other genes. The complexity degree of LASSO regression is determined by the parameter lambda (λ). The larger the λ, the greater the penalty for the linear model with more variables. A model with fewer variables should be selected. We obtained 8 candidate genes (CDKN2A, ERBB2, GRID1, NRG3, PTK6, DLC1, P4HB, and BIRC5) by LASSO regression (Fig. 4 b-c).

Multivariate Cox regression analysis of the training cohort indicated that CDKN2A, ERBB2, PTK6, and BIRC5 were independent prognostic factors according to the HR values (CDKN2A: 1.571; ERBB2: 2.310; PTK6: 1.355; and BIRC5: 2.375; *P* < 0.05) (Table 2). Therefore, these genes were used to establish a prognostic model risk score = (0.45 ∗ CDKN2A expression) + (0.84 ∗ ERBB2 expression) + (0.30 ∗ PTK6 expression) + (0.86 ∗ BIRC5 expression).

**Table 2:** The results of univariate and multivariate Cox regression analysis

Gene	Univariate		Multivariate	
	HR(95%CI)	*P*	HR(95%CI)	*P*
ERBB2	2.746 (1.616–4.665)	0.001	2.310 (1.084–4.923)	0.030
CDKN2A	1.27 (1.130–1.427)	0.000	1.571 (1.290–1.914)	0.000
BAK1	1.496 (1.109–2.019)	0.008		
GRID1	1.296 (1.022–1.644)	0.037		
NRG3	1.328 (1.127–1.564)	0.000		
PTK6	1.27 (1.073–1.504)	0.006	1.355 (1.062–1.730)	0.015
DLC1	0.550 (1.367–0.822)	0.004		
P4HB	0.626 (0.426–0.921)	0.017		
BIRC5	2.132 (1.046–4.346)	0.033	2.375 (1.147–4.917)	0.020

Regression coefficients, *P* values, HRs, and 95% confidence intervals of the prognosis-related autophagy genes are shown

We analyzed the relationship between our prognostic model and the molecular classification of endometrial cancer, and the results showed that patients with CN high have the highest risk score according to our prognostic model, but there was no significant difference in risk between POLE, MSI-H, and CN low (Supplementary Fig. S1A). We then analyzed the relationship between risk score and clinicopathological parameters in UCEC, and the results indicated that high-risk score also correlated significantly with older age, higher FIGO stage, higher neoplasm histologic grade, histological type, and higher BMI in UCEC (Supplemental Table S4). Furthermore, we have performed the COX analysis again and included risk score, age, histological type, and FIGO stage, and the results showed that, like traditional endometrial cancer prognostic markers, the prognostic score obtained by our prognostic model was also an independent prognostic factor for endometrial cancer, with an HR of 2.627 and a *p*-value less than 0.01(Supplemental Table S5, Supplemental Fig. S1B).

**Fig. 5:**
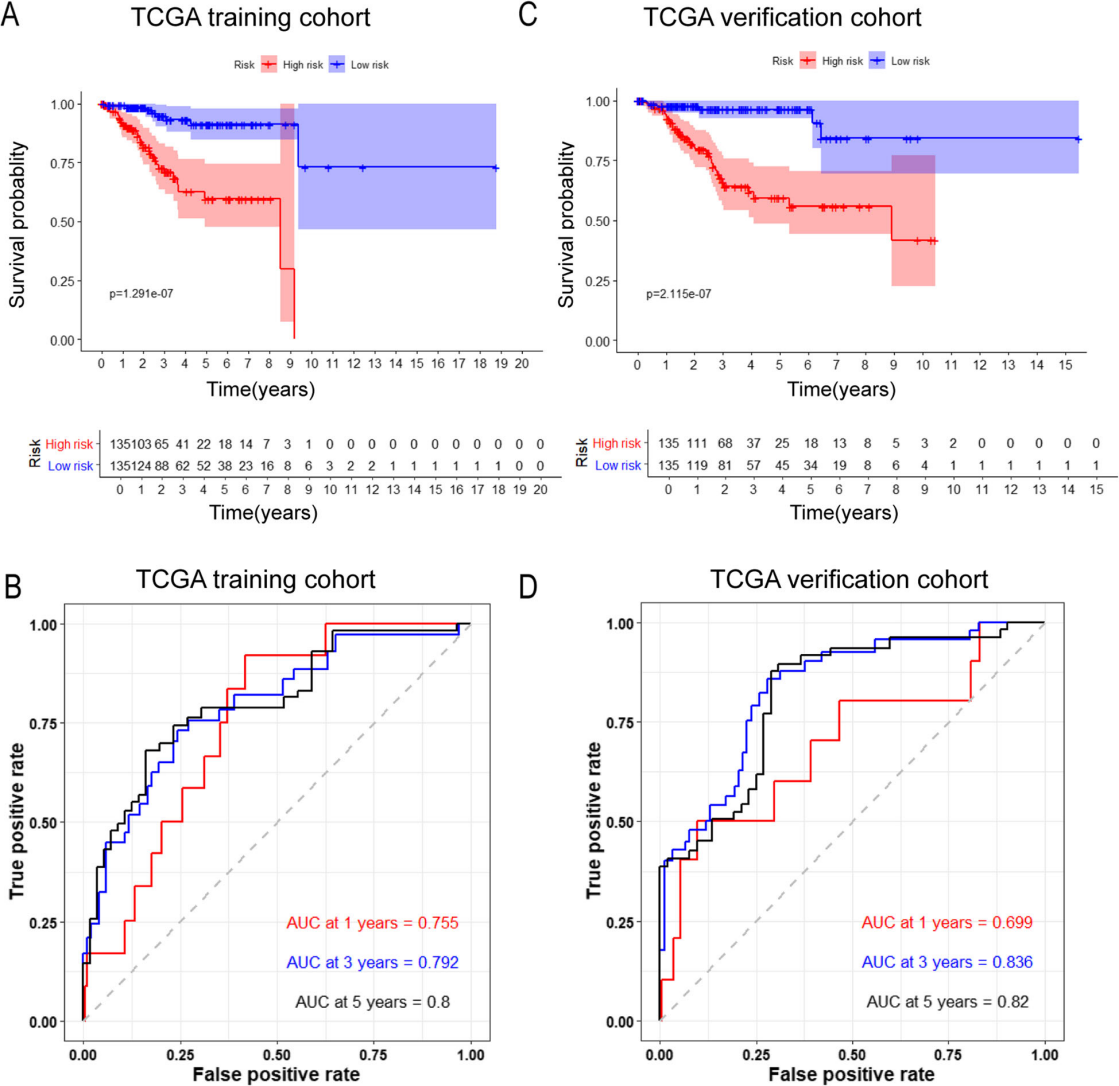
Validation of the prognostic model. **a** Kaplan-Meier plotter of the high-risk and low-risk UCEC patients in the training group; **b** Time-dependent ROC curves for predicting one-year, three-year, and five-year survival in the training cohort; **c** Kaplan-Meier plotter of the high-risk and low-risk UCEC patients in the testing group; **d** Time-dependent ROC curves for predicting one-year, three-year, and five-year survival in the testing cohort

We used the median risk value to divide the training set and the verification set into a high-risk group and a low-risk group. Kaplan–Meier plotter results showed that survival rates for high-risk patients in the training set for 1, 3, and 5 years were 76.0, 30.1, and 13.2%, respectively and survival rates for low-risk patients in the training set for 1, 3, and 5 years were 91.0, 45.6, and 27.9%, respectively (Fig. 5a). To evaluate the predictive accuracy of the prognostic model, we also plotted a ROC curve, where the area under the curve (AUC) was 0.755 for one-year survival, 0.790 for three-year survival, and 0.800 for five-year survival (Fig. 5b).

Similarly, we conducted a survival analysis in the verification set, and the results showed that survival rates for high-risk patients in the verification set for 1, 3, and 5 years were 82.2, 27.4, and 13.3%, respectively, while survival rates for low-risk patients in the verification set for 1, 3, and 5 years were 88.1, 45.2, and 25.2%, respectively (Fig. 5c). To evaluate the predictive accuracy of the prognostic model, we also plotted a ROC curve, where the AUC was 0.699 for one-year survival, 0.836 for three-year survival, and 0.820 for five-year survival (Fig. 5d). Besides, AUC curves of other risk factors were also plotted to compare the prognostic value of different criteria, and the results showed that our prognostic model was indeed better than traditional prognostic indicators in predicting the survival of patients with endometrial cancer, including clinical grade, age, etc. (Supplemental Fig. S2).

**Fig. 6:**
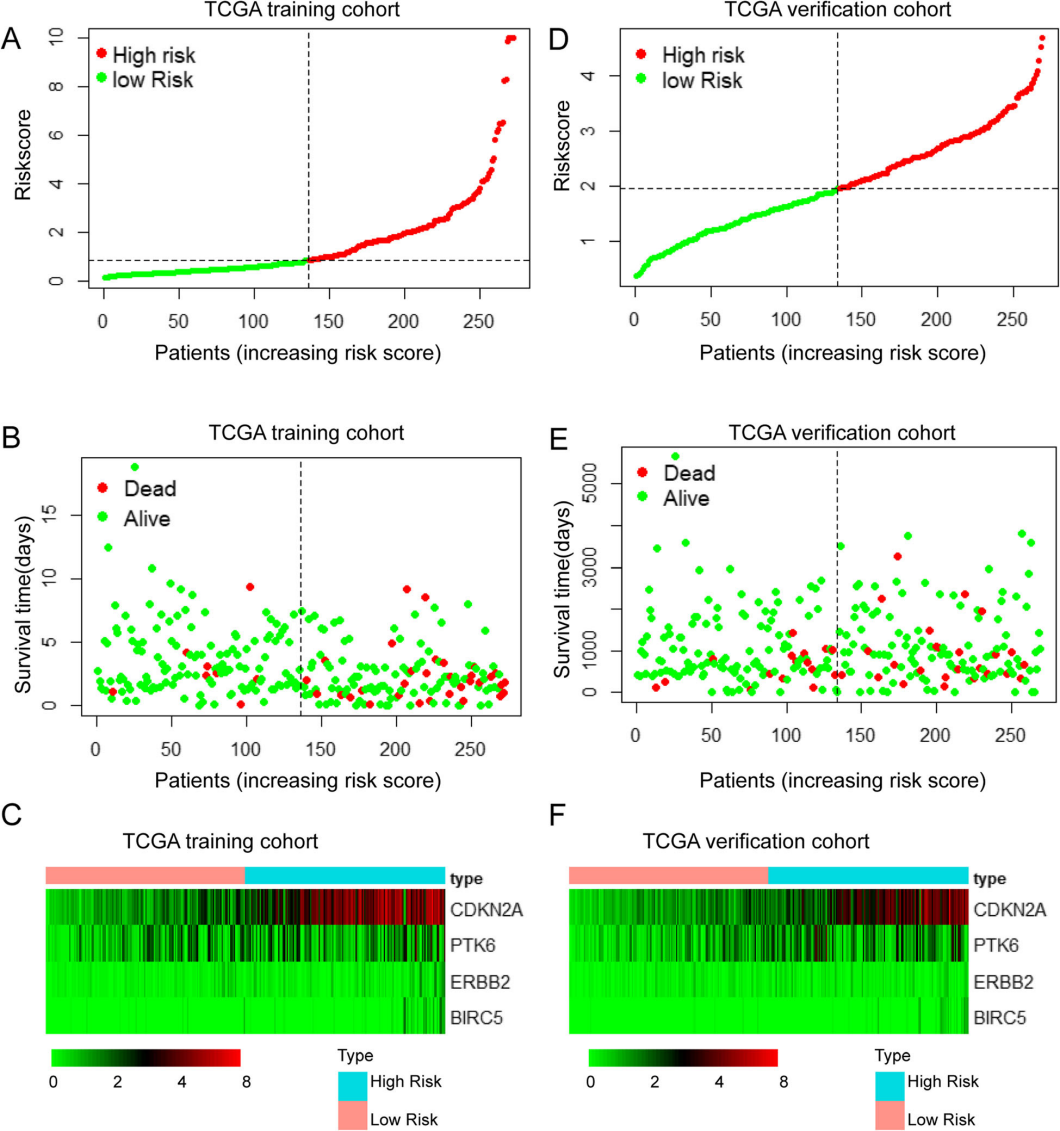
Risk curve and heatmap of risk genes in the training and verification sets. **a**, **d** Risk score distribution of UCEC patients with different risks in the training set and verification set. **b**, **e** Scatterplots of UCEC patients with different survival status in the training set and verification set. **c**, **f**) The heatmap of risk genes in UCEC between high-risk and low-risk patients in the training and verification sets

In addition to using survival curves to validate our prognostic model, we plotted risk curves for patients with endometrial cancer in the training set and the verification set. In both sets, as the patient’s risk value increased, patient mortality increased significantly. A heatmap showed expression of risk genes was up-regulated in the high-risk groups. The results from the training and verification sets were internally consistent (Fig. 6 a-f).

### Validation of risk genes at the protein level

**Fig. 7:**
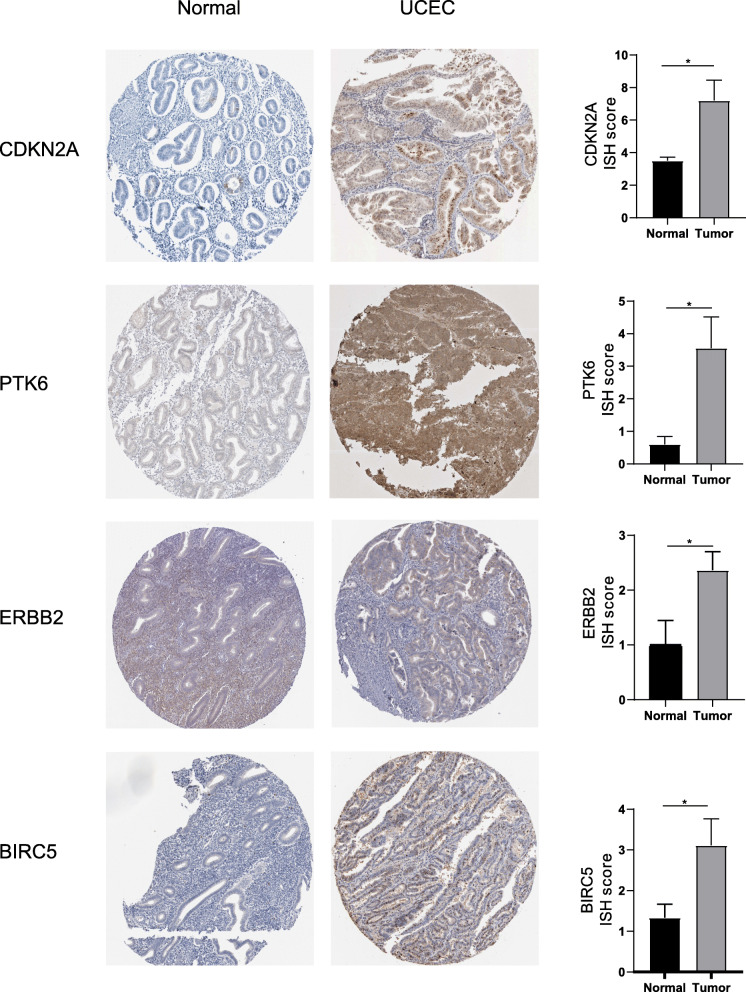
Validation of risk genes at the protein level. Immunohistochemical staining of the four risk ARGs in normal endometrium and endometrial cancer tissues (left) and a histogram showing the staining scores for normal endometrium and endometrial cancer tissues (right). *P < 0.05. NS: not significant

Immunohistochemistry revealed ISH scores for four risk ARGs (CDKN2A, ERBB2, PTK6, and BRIC5) were significantly higher in endometrial cancer tissue than in healthy endometrial tissue, which suggested that these genes are highly expressed in endometrial cancer tissues. We also found that CDKN2A was mainly located in the cytoplasm, membrane, and nucleus, while ERBB2, BRIC5, and PTK6 were mainly located in the cytoplasm and membrane. All immunohistochemical results were derived from the HPA database (Fig. 7), the corresponding data are referenced on Table S3.

## Discussion

Endometrial cancer is the fourth most common malignant tumor in women worldwide, and its incidence is still increasing in recent years [25]. Although most patients with endometrial cancer are postmenopausal women, the incidence in young women has dramatically increased as a result of early-onset obesity and hyperinsulinemia. It is speculated that one-third of women will suffer from endometrial cancer during their lifetime [26, 27].

The role of autophagy in tumor progression is not yet clear. It has been reported that in the early stages of tumorigenesis, autophagy can inhibit tumor growth by inhibiting tumor necrosis, and as the tumor continues to develop, autophagy protects tumors by promoting tumor vascularization and distant metastasis [28]. In the field of endometrial cancer research, it has been reported that isoliquiritigenin (ISL) can induce autophagy in endometrial cancer cells, thereby exerting a cancer-suppressing effect [29]. High levels of estrogen affect glutathione metabolism by increasing the level of glutaminase, thereby inhibiting autophagy in endometrial cancer and promoting its growth [30]. Kanda R, et al. showed that glucagon-like peptide-1 receptor (GLP-1R) promotes autophagy in endometrial cancer, thereby inhibiting its growth and suggesting GLP-1R agonists as a potential therapeutic strategy [31].

Previous studies have mostly focused on the mechanism of autophagy in regulating tumorigenesis and the development of endometrial cancer. In this study, we sorted and analyzed the transcript information of endometrial cancer samples retrieved from TCGA database and screened out 37 differentially expressed ARGs. GO enrichment analysis found that most of these genes are part of the intrinsic apoptotic signaling pathway, processes utilizing the autophagic mechanism, and cellular responses to oxidative stress, which are important functions of autophagy. The autophagy process can remove abnormal proteins and damaged organelles produced by cells under oxidative stress, thus becoming an important guarantee for the health and survival of cells. Previous studies reported that autophagy can promote mammalian cell survival by inhibiting oxidative stress and TP53 function [32, 33]. KEGG analysis found that most of these genes are mainly involved in autophagy, apoptosis, the HIF-1, and ERBB signaling pathways. Activation of autophagy promotes tumor metastasis by inducing HIF-1α, and it was verified that HIF-1 modulates the induction of autophagic proteins through regulating the association between Beclin1 and Bcl-2 [34, 35]. The ERBB receptors transduce signals through Akt, MAPK, and many other pathways to regulate cell proliferation, migration, differentiation, and apoptosis. It was reported that endogenous HER2 interacts with Beclin1 in breast cancer cells to inhibit autophagy, and that Beclin1 mutation reduces Beclin1/HER2 binding to promote autophagy in HER2-positive breast cancer cells [36].

We constructed a prognostic model of endometrial cancer containing four risk ARGs (CDKN2A, PTK6, ERBB2, and BIRC5) using multivariate and univariate Cox, and LASSO regression analyses. By performing survival analysis on the training set and test set and drawing ROC curves, we proved that our model can predict the prognosis for endometrial cancer patients based on risk factors and that the accuracy of the model was relatively high.

CDKN2A contains two introns and three exons and generates several transcript variants, which differ in their first exons, including the p16^INK4a^ and P14^ARF^ proteins, which are negative regulators of the cell cycle [37–39]. The expression of the P14^ARF^ gene in endometrial cancer is significantly higher than in normal endometrium, but the relationship between P14^ARF^ and endometrial cancer prognoses was not investigated [40]. A previous study reported that CDK inhibitors induce cytotoxicity by enhancing apoptosis in CDKN2A-defective SqCLC cells, resulting in increased autophagy during this process [41]. Furthermore, García-Prat L et al. reported that monoubiquitination of lysine 119 (H2Aub) at the INK4a locus drives p16^INK4a^ induction in geriatric satellite cells, which promotes autophagy [42]. Capparelli C et al. found that p16^INK4a^-overexpressing fibroblast cell lines significantly promoted tumor growth and the expression of p16^INK4a^ was associated with the induction of markers of senescence, autophagy, and mitophagy in epithelial cancer cells [43]. Moreover, Witkiewicz AK et al. reported that p16^INK4a^ was elevated in ductal carcinoma in situ and stoma of breast cancer, and that elevated p16^INK4a^ expression was closely related to disease recurrence [44]. Furthermore, CDKN2A was thought to inhibit tumor growth [41]; however, the mechanisms that promote tumor progression remain unclear. In the present study, we identified elevated CDKN2A expression as an independent risk factor for endometrial cancer, with higher levels of CDKN2A expression corresponding with worse prognosis for patients with endometrial cancer. A possible explanation for this is that CDKN2A overexpression in the stroma might affect the tumor microenvironment through the secretion of cytokines and proteases and promote the growth of endometrial cancer [45, 46].

PTK6 encodes a cytoplasmic non-receptor protein kinase. PTK6 antagonists can inhibit metastasis in triple-negative breast cancer and PTK6 activation can promote epithelial-to-mesenchymal transition in prostate cancer [47]. PTEN suppresses oncogenic signaling for prostate cancer through dephosphorylation of PTK6 tyrosine 342(PY342), and PTK6 facilitates cell migration and proliferation by phosphorylating Esp8 [48]. PTK6 is also involved in autophagy related signaling pathways, and there are reports that PTK6 can promote ERBB2-induced mammary gland tumorigenesis. Additionally, STAT3, FAK, and BCAR1 are relevant PTK6 substrates in breast cancer, and PTK6 protects breast cancer cells from autophagic cell death induced by loss of anchorage, suggesting that PTK6 can inhibit autophagic processes [49]. In the present study, we found that elevated PTK6 expression was associated with worse prognoses, suggesting that PTK6 might promote endometrial cancer progression by inhibiting autophagy.

ERBB2, also known as HER2 in humans, encodes a member of the epidermal growth factor (EGF) receptor family of receptor tyrosine kinases. The overexpression of the HER2 protein was seen in approximately 25–30% of breast and ovarian cancers [50]. In high-grade endometrial cancer, the HER2 gene is amplified by17–30% and up to 80% of tumors exhibit HER2 protein overexpression [51, 52]. HER2 is an important signaling pathway in tumor growth and involved in regulating autophagy, blockage of HER2/Beclin1 binding increases autophagy, with a previous study finding that HER2 interacts with Beclin1 in breast cancer cells and inhibits autophagy [36]. Moreover, HER2-targeted therapy induces autophagy in esophageal cancer, and autophagy inhibitors significantly reduce resistance to the HER2 inhibitor lapatinib [53]. Furthermore, a previous study reported that protective autophagy promotes the resistance of HER2-positive breast cancer cells to lapatinib, suggesting that HER2 regulates protective autophagy [54, 55]. In the present study, we identified HER2 as an independent risk factor for endometrial cancer through multivariate Cox regression analysis (HER2 HR: 2.3), suggesting that HER2 might promote the progression of endometrial cancer by inhibiting autophagy.

BIRC5 is a member of the inhibitor of apoptosis gene family, which encode negative regulatory proteins that prevent apoptotic cell death. A previous study found that miR-203 inhibits ovarian tumor metastasis by targeting BIRC5/survivin and that BIRC5/survivin can also serve as a target for glycolysis inhibition in high-stage neuroblastoma [56]. Yu X, et al. reported BIRC5 overexpression in non-small cell lung cancer (NSCLC), and that miR-195 targets BIRC5 to induce apoptosis and senescence in NSCLC cells [57]. Lin et al. demonstrated that BIRC5 directly regulates autophagy by modulating the protein stability of ATG7 and physically binding to the ATG12–ATG5 conjugate. Additionally, BIRC5 negatively modulates the protein stability of ATG7 and physically binds to the ATG12–ATG5 conjugate, thereby preventing formation of the ATG12–ATG5–ATG16L1 protein complex in human cancer and suggesting that BIRC5 can directly regulate autophagy in cancer cells [58]. In the present study, we found that BIRC5 was significantly overexpressed in endometrial cancer and an independent prognostic factor, suggesting that BIRC5 might promote endometrial cancer by inhibiting autophagy in cancer cells.

A limitation of our study is that it is mainly based on data from TCGA and human autophagy databases. ARGs mechanism in promoting the occurrence and progression of endometrial cancer has not yet been elucidated. Further research and experimental studies are needed to verify whether the progress of endometrial cancer can be suppressed by downregulating these ARGs.

## Conclusion

In summary, by collecting and analyzing the transcript information of endometrial cancer samples from TCGA database, we identified four prognosis-associated autophagy genes (CDKN2A, PTK6, ERBB2, and BIRC5). These genes have potential as new biomarkers or therapeutic targets; however, their effects and mechanism of action need further experimental verification prior to clinical implementation.

## Supplementary information

**Additional file 1.** Table S1. List of differentially expressed ARGs between EC tissue and normal endometrial tissue in the TCGA dataset.

**Additional file 2.** Table S2.Gene ontology enrichment analysis of the differentially expressed ARGs in the TCGA dataset.

**Additional file 3.** Table S3. Immunohistochemical data of four risk ARGs.

**Additional file 4.** Table S4. Clinical and pathological characteristics of high and low risk patients.

**Additional file 5.** Table S5. COX analysis of age, histological type, FIGO stage and risk score.

**Additional file 6 Supplementary Figure S1**.(A) Distribution of risk score among four different subtypes of endometrial cancer, POLE ultramutated, microsatellite instability hypermutated, copy-number low, and copy-number high.(B) Forest plots visualizing the HRs of clinicopathological criteria identified by multivariate Cox analysis.

**Additional file 7 Supplementary Figure S2**.(A-D) Time-dependent ROC curves of different clinicopathological criteria for predicting one-year, three-year, and five-year survival of endometrial cancer.

## Data Availability

All analyzed data are included in this published article and its supplementary information file. The original data are available from the corresponding author on. reasonable request.

## Abbreviations

UCEC: Uterine Corpus Endometrial Carcinoma
TCGA: The Cancer Genome Atlas
ARGs: autophagy related genes
CI: Confidence interval
OS: Overall survival
ROC: Receiver operating characteristic
GO: Gene Ontology
KEGG: Kyoto Encyclopedia of Genes and Genomes
LASSO: Least Absolute Shrinkage and Selection Operator
HR: hazard ratio
HPA: Human Protein Atlas
ISH: Immunohistochemistry
AUC: Area under the curve
