# Supplementary figures and images for: An integrated autophagy-related gene signature predicts prognosis in human endometrial Cancer
Source: BMC Cancer. 2020 Oct 27;20:1030. doi: 10.1186/s12885-020-07535-4 (PMC7590615; doi:10.1186/s12885-020-07535-4)

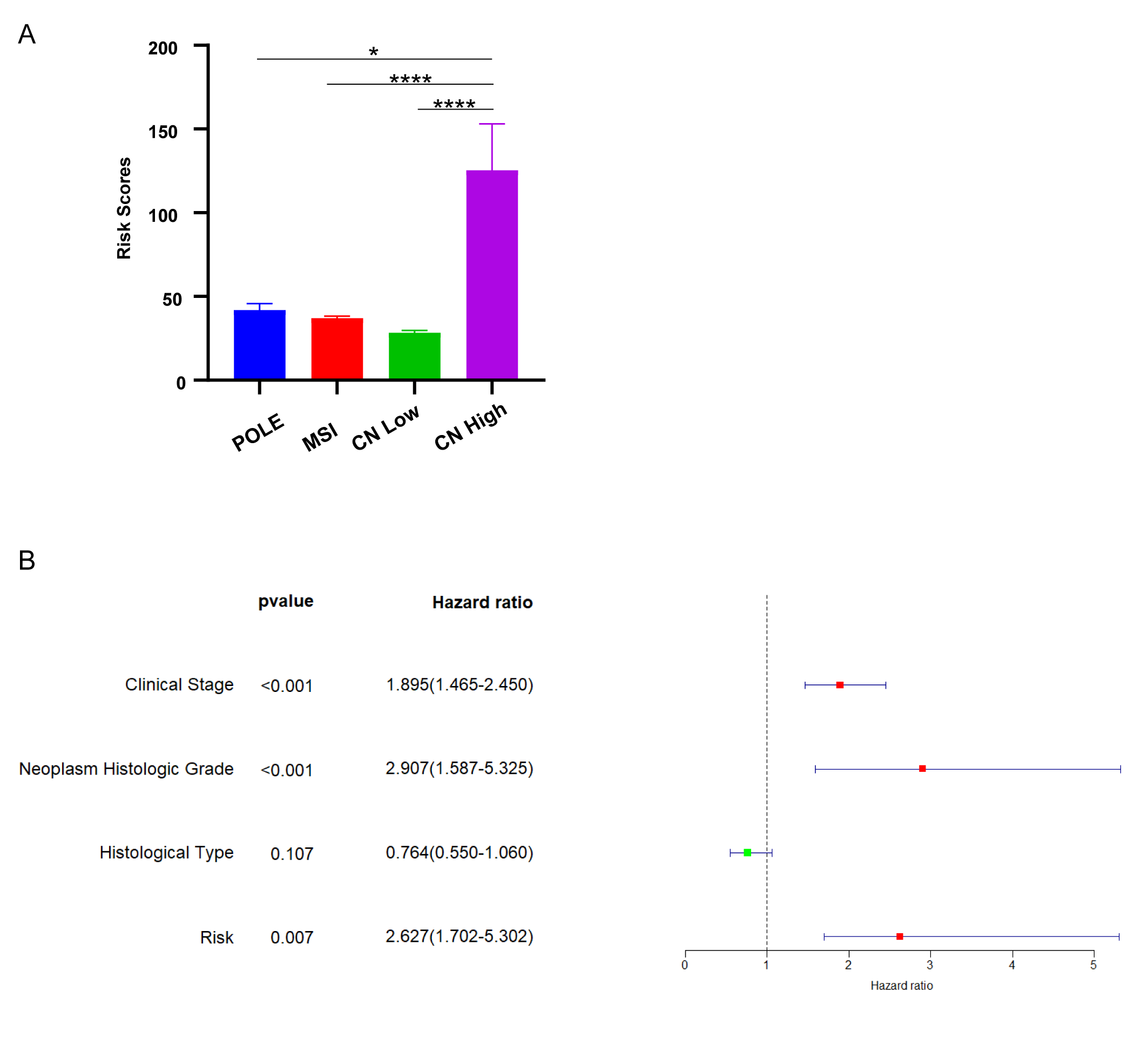

Supplement: Supplementary file 6 — Additional file 6 Supplementary Figure S1.(A) Distribution of risk score among four different subtypes of endometrial cancer, POLE ultramutated, microsatellite instability hypermutated, copy-number low, and copy-number high.(B) Forest plots visualizing the HRs of clinicopathological criteria identified by multivariate Cox analysis. [file 12885_2020_7535_MOESM6_ESM.tif]

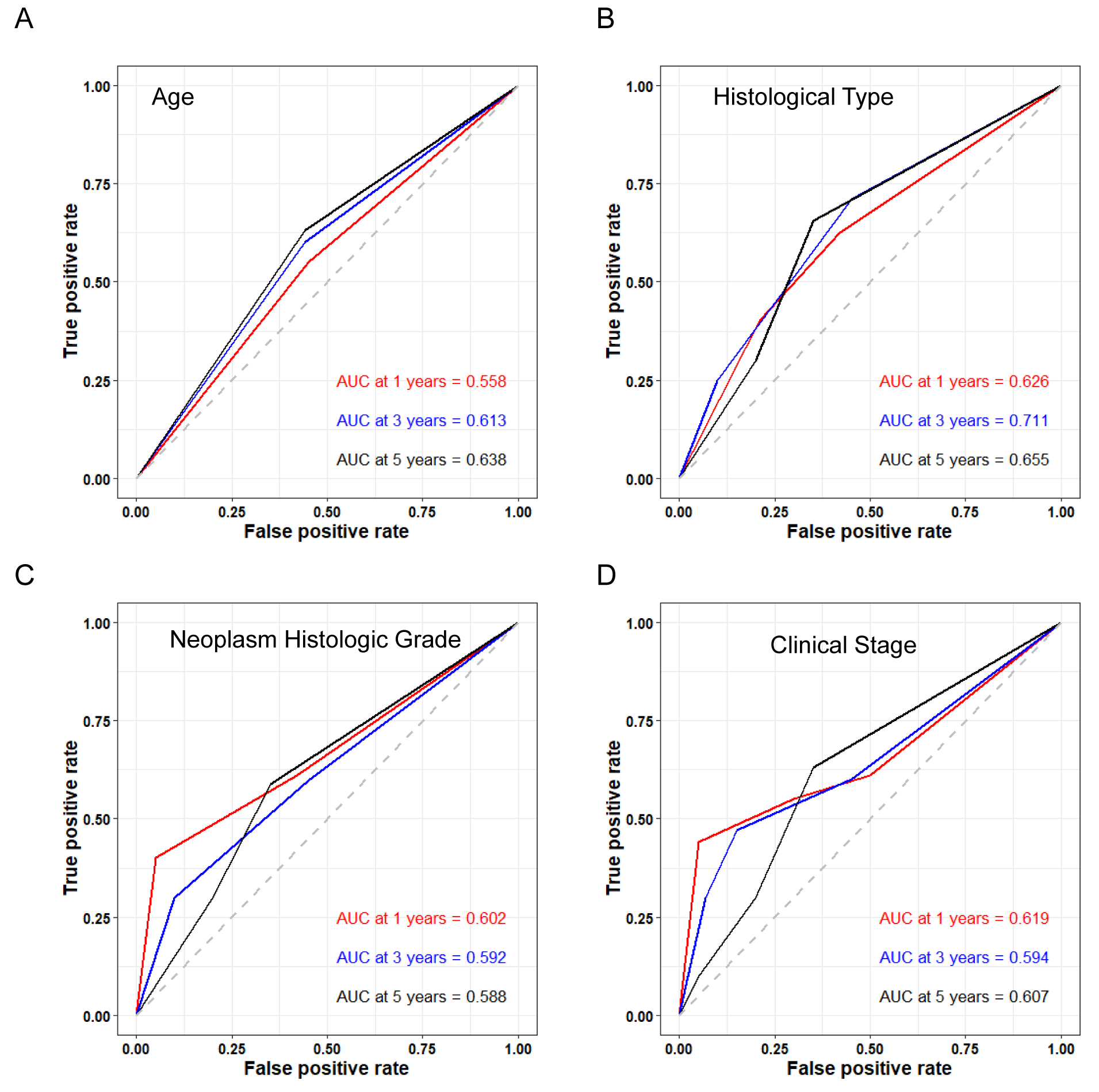

Supplement: Supplementary file 7 — Additional file 7 Supplementary Figure S2.(A-D) Time-dependent ROC curves of different clinicopathological criteria for predicting one-year, three-year, and five-year survival of endometrial cancer. [file 12885_2020_7535_MOESM7_ESM.tif]
